# American Pharmaceutical Association

**Published:** 1877-10

**Authors:** 


### Editorial.

---

The following abstract of proceedings had in the American Pharmaceutical Association, held at Toronto, Canada, as reported to the *Virginia Medical Monthly*, shows increasing interest felt in the progress of pharmacy. Though taught and practiced as a separate science, the operations of pharmacy are so intimately connected with, and so essential to, the medical profession, that advancement in knowledge on this subject should be hailed as progress in medicine. The next meeting of the Association, as will be seen below, convenes in Atlanta, Ga., on the first Tuesday in September, 1878:

American Pharmaceutical Association.—Thinking that a short letter detailing some of the proceedings of the "American Pharmaceutical Association," now in session in this city, (Toronto,, Canada,) would be interesting to your readers, I have concluded to drop you a few lines.

The 25th annual meeting of this Association (which has on its roll over 1,000 members) was called to order in the City Council Chamber, by the President, Mr. Bullock, of Philadelphia, at 3 o'clock, p.m., Tuesday, September 3d, 1877. The mayor being absent from the city, Alderman Wright represented him, and made a short address of welcome, which was replied to by Mr. Bullock. About one hundred delegates and members were present.

After some preliminaries of organization, the President read his annual address. Commencing with a reference to the fact that this Association had just reached the first quarter of a century of its history, and alluding to its large membership, and also to the fact that this was the first time that they had ever met outside of the boundaries of the United States, he passed on to a review of the history and progress of pharmacy, tracing it back to its earliest beginning.

The reports of the Permanent Secretary, Prof. J. F. Maisch, and of the chairman of the Executive Committee, both very interesting to pharmacists, were then read. Some invitations to entertainments presented from the Ontario College of Pharmacy and Toronto druggists, were read and accepted; and this about finished the business of the first session.

Owing to the great distance, and the expense involved, the Southern States were not well represented. South Carolina had only one representative (Mr. Heinith, of Columbia); Virginia two (F. H. Masi, of Norfolk, and T. R. Baker, of Richmond); Georgia three (Mr. Tarrant, of Augusta, and Mr. Ingalls and Mr. Menard, of Macon); and Kentucky only one (Prof. Diehl, of Louisville). The Western States did not turn out well, but the Northern, Eastern and Middle Atlantic States were well represented.

To those who have yet to visit Toronto, I will say they may be prepared to see a very handsome city, well built, and regularly laid out. The public buildings are massive, very large, and very fine specimens of architecture.

At the opening session of the Association, a resolution was passed, inviting the faculty of the University of Toronto, and the medical profession of the city, to attend the sessions of the Association.

At the second session of the Association, the nominating committee reported the names of officers for the ensuing year, which were duly elected. Mr. W. Saunders, of London, Ontario, was elected President for the ensuing year; Mr. Ewen McIntire, of New York, first Vice President; Mr. John Ingalls, of Georgia, second Vice President, and Mr. Emlen Painter, of San Francisco, third Vice President.

The standing committees, such as the Committee on the Drug Market, on Papers and Queries, on Prize Essays, on Legislation, etc., were all reorganized as usual.

Dr. Wilson H. Pile, of Philadelphia, Chairman of Committee on Adulterations and Sophistications, read a very interesting report.

By-the-way, on the morning before the final adjournment of the meeting, at 4 o'clock, this old gentleman was visited with a stroke of paralysis, which rendered him helpless, but not speechless, and his calls for assistance could only be responded to by getting through the opening over the top of his chamber door. The physician who was summoned to attend him said he would recover.

On the second day the Mayor, who had returned to the city, entered the hall, amidst great applause, and proceeded to address the meeting, apologizing for his unavoidable absence from town at the time of the opening of the meeting, tendering the hospitalities of the city, etc. After wishing the members of the Association a pleasant meeting, he retired.

Considerable time was occupied at all the sessions of the Convention in reading essays, replies to queries, and special reports, and in discussions thereon. The subject of the revision of the Pharmacopœia, which you remember was tabled at the last meeting of the American Medical Association, came up yesterday in the regular order of business. Dr. Squibb made a few ramarks concerning his connection with the subject. He had advocated a revision of the Pharmacopœia in the past, but now he was content to let the matter rest. He would be satisfied with any pharmacopœia adopted by the Association. He said his efforts in behalf of a new pharmacopœia had proceeded from the purest motives, and his motives had been misconstrued and himself abused until he was disgusted, and would have nothing more to do with it. Mr. Sheppard, of Boston, rose and said, that from his youth he had learned to look upon Dr. Squibb as high authority, both in medicine and pharmacy, and to honor him for his great integrity of character. He then offered the following resolution, which was unanimously agreed to, viz: "*Resolved*, That while there is among the members of the American Pharmaceutical Association an honest difference of opinion as to the advisability of the plan suggested by Dr. Squibb for the revision of the U. S. Pharmacopœia, the thanks of the Association be, and are hereby tendered to Dr. E. R. Squibb, of Brooklyn, N. Y., for his earnest efforts during the past two or three years to inaugurate an improvement in the plan of revision of the U. S. Pharmacopœia." Dr. Squibb rose and gracefully thanked the Association for the compliment paid him in this resolution.

A committee of five was then appointed to consider and report a plan of action with reference to the revision of the Pharmacopœia. The committee recommended that a committee of fifteen be appointed, to be selected by the President, with special reference to their competency, whose business it shall be to revise the present Pharmacopœia, and make a new one, to be presented at the next meeting of the Association; and if approved, then to be presented to the American Medical Association, subject to their approval, and open to any changes which they may think proper to suggest—the sole object of the pharmaceutical committee being to do the work as they (the physicians) want it done. It is understood that Dr. Squibb declined to let his name be placed on the committee. An able committee, however, was appointed, and one that will work with energy and enthusiasm, dividing themselves into sub-committees, and appropriating the respective branches, such as materia medica, botany, chemistry and pharmacy, to those most familiar with the respective subjects.

When the subject of the next place of meeting came up, Mr. Ingalls, of Macon, Ga., rose and extended an invitation to the Association to meet in Atlanta, Ga. This proposition was immediately seconded by all the Southern members present, who promised their Northern brethren a cordial and hospitable reception. The committee appointed to recommend the next place of meeting reported unanimously in favor of Atlanta, Ga., and when the Association adjourns it will do so to meet in Atlanta, Ga., on the 1st Tuesday in September, 1878.

The Canadians are entertaining their foreign visitors in a very cordial spirit of genuine hospitality, and have on several occasions provided long processions of fine carriages, in which they took the members of the Assosiation, and the ladies accompanying them, through the most beautiful and attractive portions of the city, occasionally stopping to vacate the carriages, and guide their guests through some of the handsome public parks and buildings which adorn the upper part of the city.

But I fear that, unless I cease firing, so to speak, I will contradict the proposition which heads this letter; viz., to write a *short letter.*

